# Supplementary material for: Solvent-derived defects suppress adsorption in MOF-74
Source: Nat Commun. 2023 Apr 25;14:2386. doi: 10.1038/s41467-023-38155-8 (PMC10130178; doi:10.1038/s41467-023-38155-8)
Supplement: Supplementary file 1 — Supplementary Information [file 41467_2023_38155_MOESM1_ESM.pdf]

Supplementary Information

Solvent-derived Defects Suppress Adsorption in MOF-74

Yao Fu, Yifeng Yao, Alexander C. Forse, Jianhua Li, Kenji Mochizuki, Jeffrey R. Long, Jeffrey A. Reimer, Gaël De Paëpe, Xueqian Kong*

Correspondence to: kxq@zju.edu.cn

This PDF file includes:

Supplementary Tables 1 to 2
Supplementary Figures 1 to 17

Supplementary Table 1. Quantities of $Mg(NO_3)_2 \cdot 6H_2O$ used in the synthesis of ideal and defective Mg-MOF-74 samples.

Sample	Amount of $Mg(NO_3)_2 \cdot 6H_2O$ used in synthesis
**Ideal**	0.143 g (0.559 mmol)
**0.03D**	0.215 g (0.838 mmol)
**0.08D**	0.286 g (1.117 mmol)
**0.16D**	0.430 g (1.676 mmol)
**0.23D**	0.573 g (2.235 mmol)
**0.30D**	0.857g (3.352 mmol)
**0.36D**	1.146 g (4.469 mmol)
**0.41D**	1.576 g (6.145 mmol)

Supplementary Table 2. The concentration ratios of different chemical constituents in Mg-MOF-74. The concentration of dobpdc^{4-} linker ($[\mathrm{dobdc}^{4-}]$) and the concentration of formate ($[\mathrm{For}^{-}]$) are obtained from solution-state ^{1}H NMR. The concentration of Mg^{2+} ($[\mathrm{Mg}^{2+}]$) is determined by ICP-OES.

Sample	$\frac{[\mathrm{For}^{-}]}{[\mathrm{dobdc}^{4-}]}$	$\frac{[\mathrm{dobdc}^{4-}]}{[\mathrm{Mg}^{2+}]}$	$\frac{[\mathrm{For}^{-}]}{[\mathrm{Mg}^{2+}]}$	$\frac{4\times[\mathrm{dobdc}^{4-}]\ +1\times[\mathrm{For}^{-}]}{4\times[\mathrm{Mg}^{2+}]}$
**Ideal**	0	0.51	0.00	1.02
**0.03D**	0.13	0.48	0.06	0.98
**0.08D**	0.37	0.47	0.17	1.03
**0.16D**	0.77	0.41	0.32	0.98
**0.23D**	1.18	0.38	0.45	0.98
**0.30D**	1.74	0.35	0.61	1.01
**0.36D**	2.21	0.33	0.73	1.02
**0.41D**	2.80	0.30	0.85	1.03

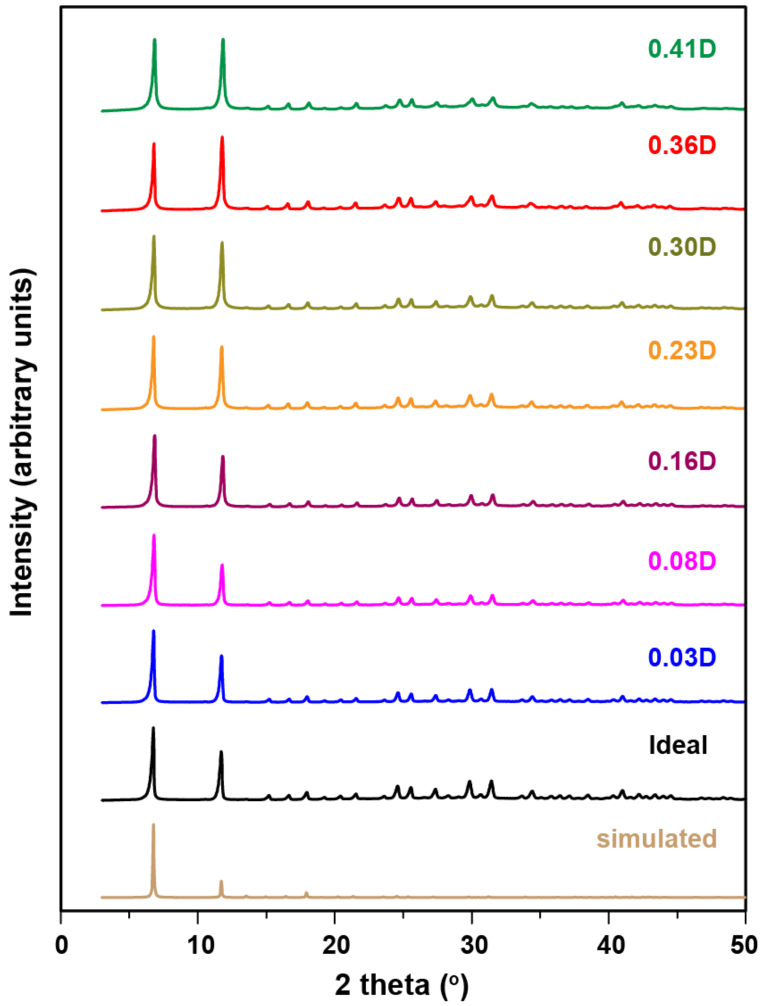

Supplementary Figure 1. Powder XRD patterns obtained on as-synthesized Mg-MOF-74 samples.

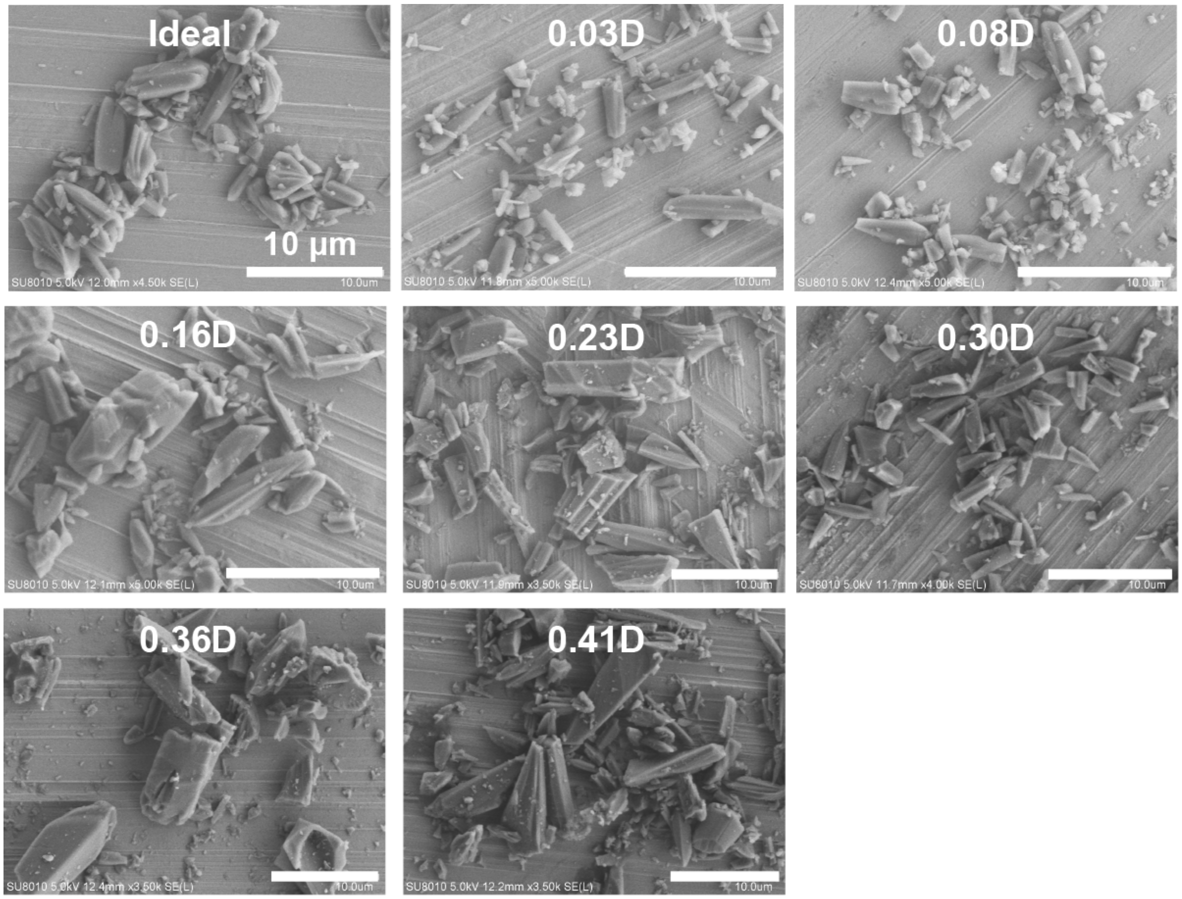

Supplementary Figure 2. SEM images of Mg-MOF-74 samples with various defect concentrations. Scale bar:10 μm (for all images).

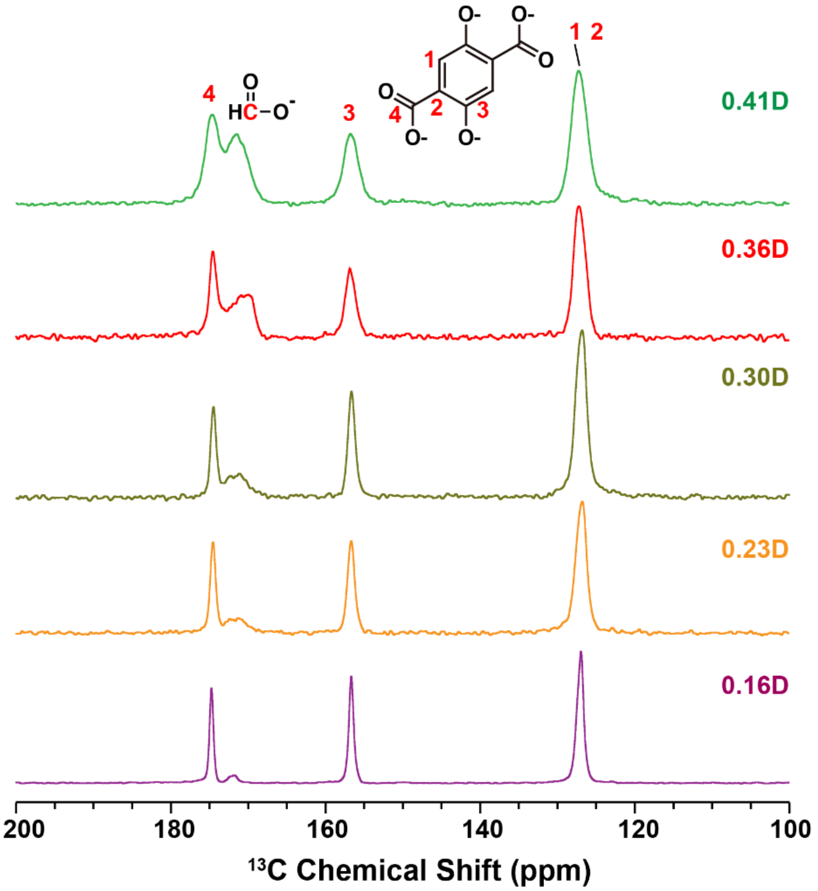

Supplementary Figure 3. ^{13}C CPMAS spectra with long contact time (CT=3000-4000 μs) of as-synthesized Mg-MOF-74 samples. The ^{13}C peaks of dobdc^{4-} linker broaden in MOFs with higher concentration of defects suggesting the increase of local disorder.

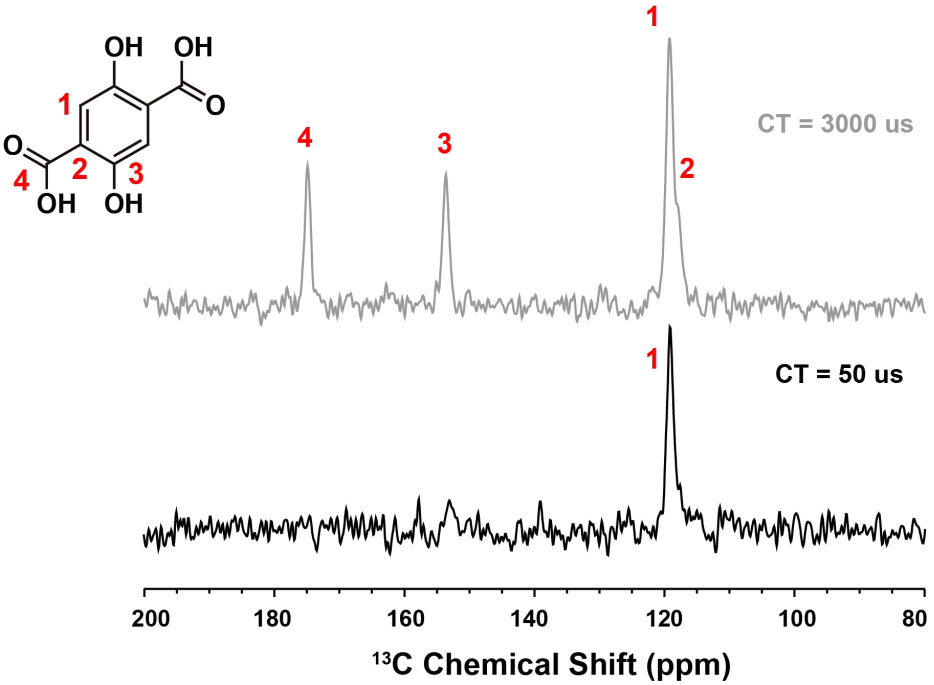

Supplementary Figure 4. ^{13}C CPMAS spectra with long contact time (CT = 3000 μs) and short contact time (CT = 50 μs) on pure H_4dobdc linker. Only the aromatic ^{13}C signal "1" can be observed at the short contact time.

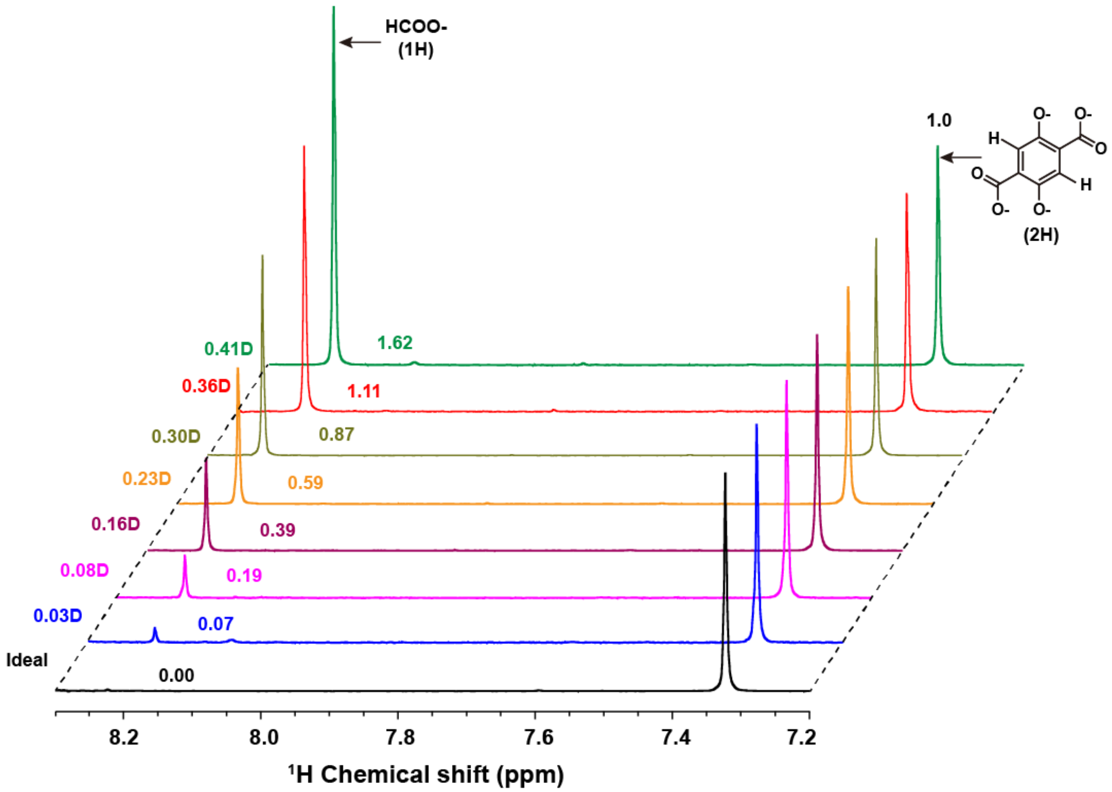

Supplementary Figure 5. ^{1}H NMR spectra of dissolved Mg-MOF-74 samples. The intensity at 8.2 ppm increases for the samples with increasing defect concentrations.

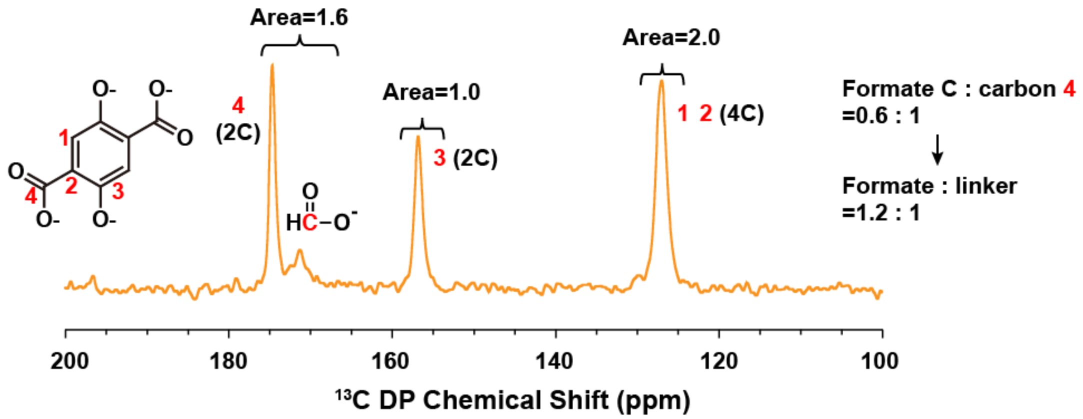

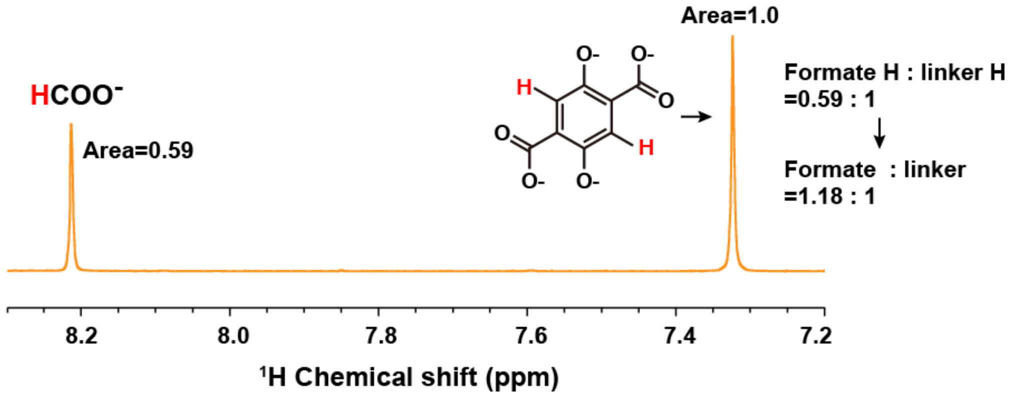

Supplementary Figure 6. Top: ^{13}C direct polarization (DP) solid-state NMR on 0.23D sample, which quantifies the formate to linker ratio = 1.20 : 1; Bottom: ^{1}H solution-state NMR of digested 0.23D sample, which quantifies the formate to linker ratio = 1.18 :1.

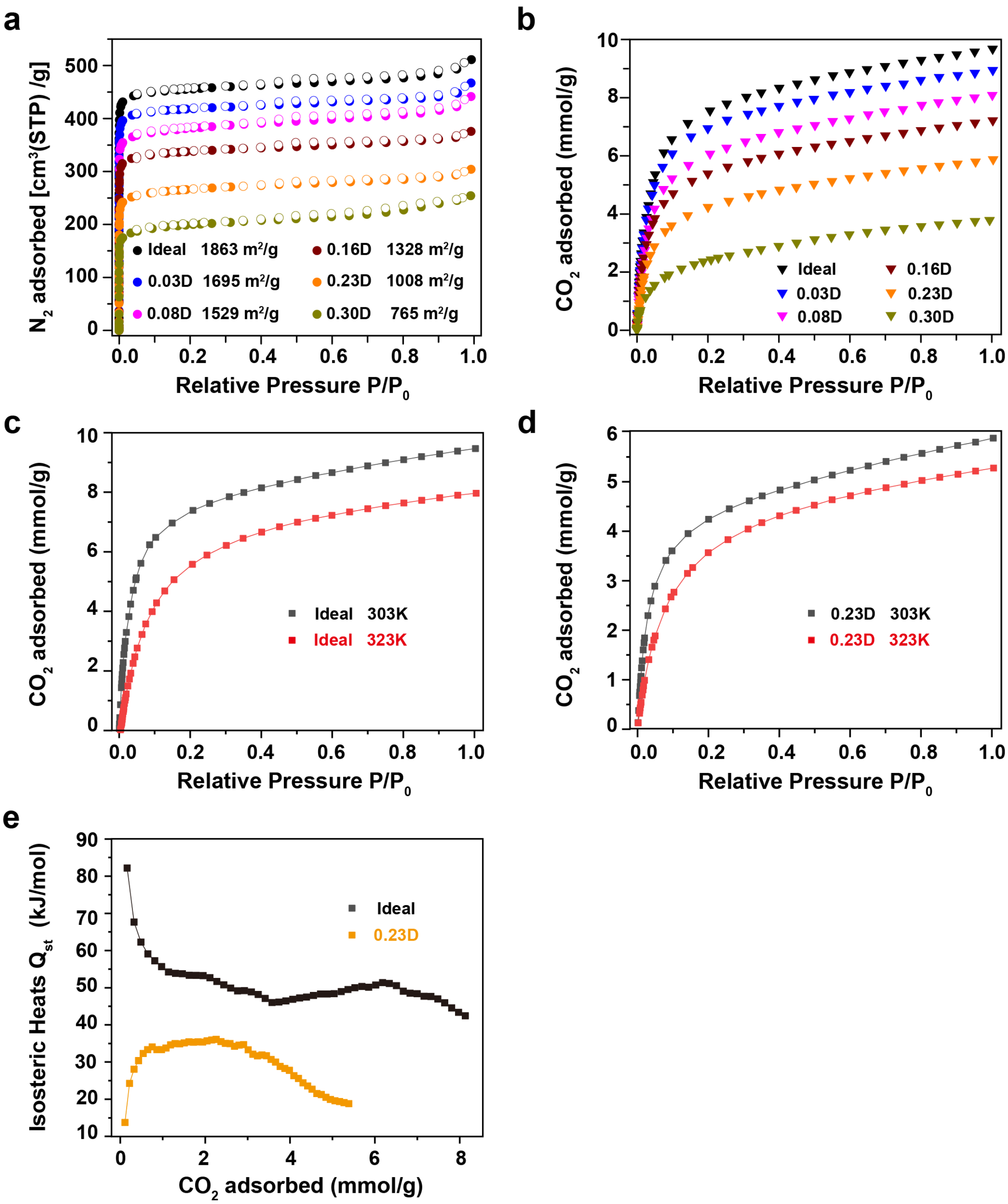

Supplementary Figure 7. (a) N_2 adsorption isotherms measured at 77 K on samples activated under vacuum at 523 K. The BET surface areas are shown. The adsorption is shown as solid circles, and the desorption is shown as open circles. (b) CO_2 adsorption isotherms measured at 303 K on samples activated under vacuum at 523 K. CO_2 adsorption isotherms measured at 303 K and 323 K on (c) ideal sample and (d) 0.23D sample; and their corresponding isosteric heats of adsorption (e).

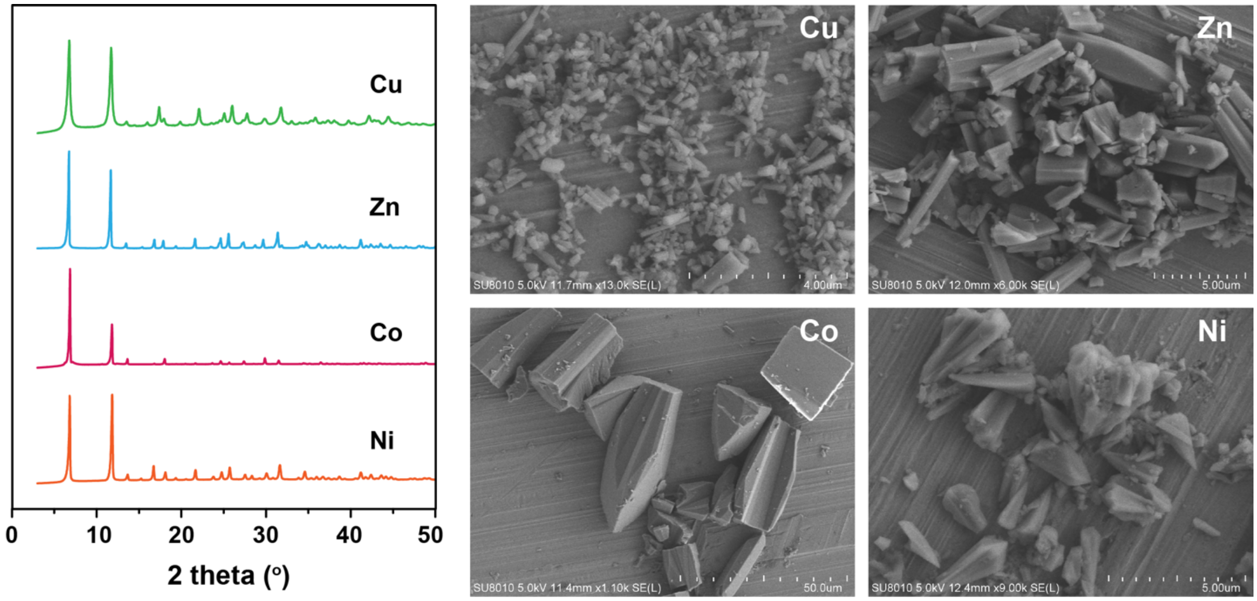

Supplementary Figure 8. PXRD patterns and SEM images of MOF-74 samples composed of Cu, Zn, Co and Ni metal ions. The metal-to-linker ratios are 6:1 in the precursor solutions.

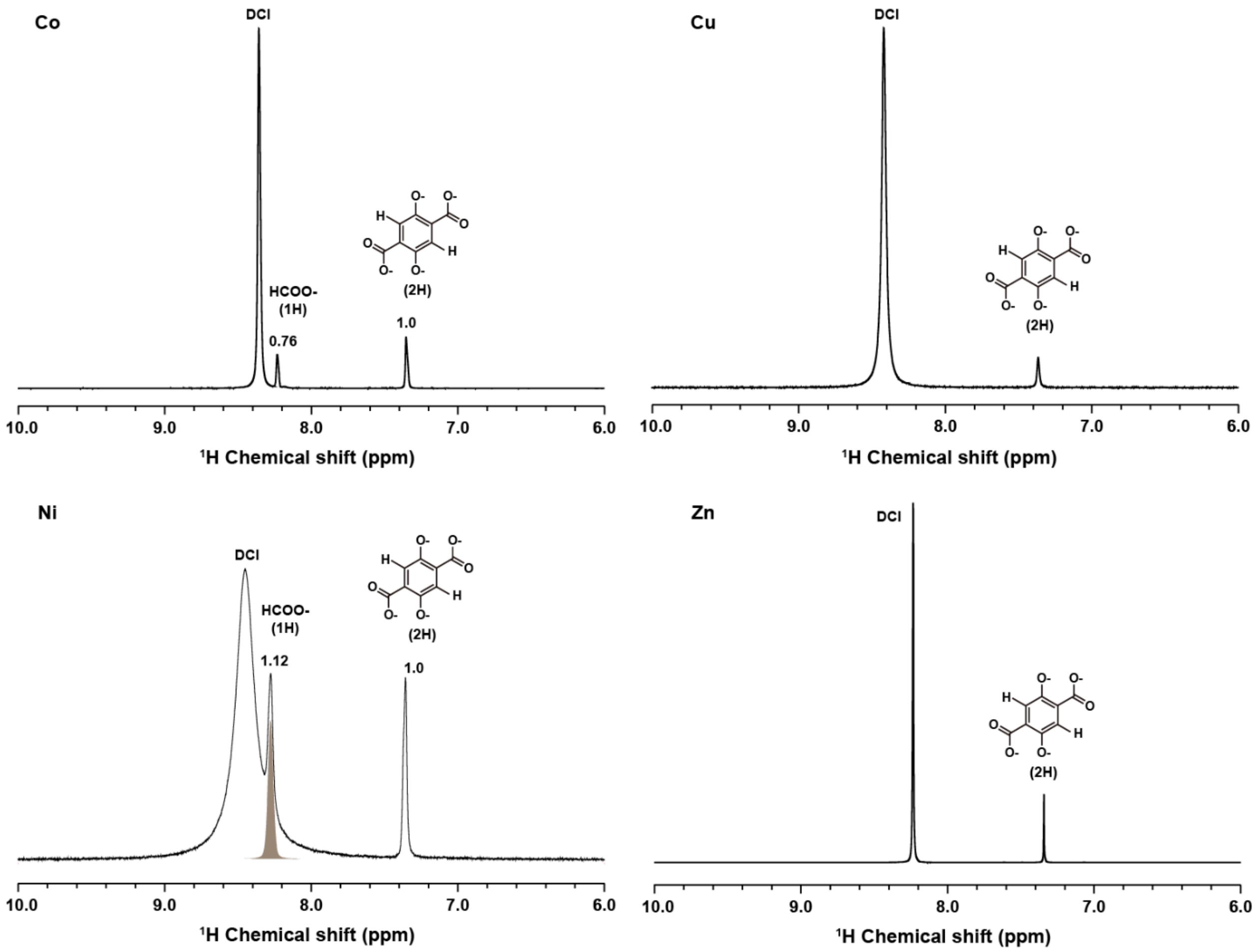

Supplementary Figure 9. ^{1}H solution-state NMR spectra of digested MOF-74 samples composed of Cu, Zn, Co and Ni metal ions. The metal-to-linker ratios are 6:1 in the precursor solutions. Formate signals are observed in the digested samples of Co-MOF-74 and Ni-MOF-74.

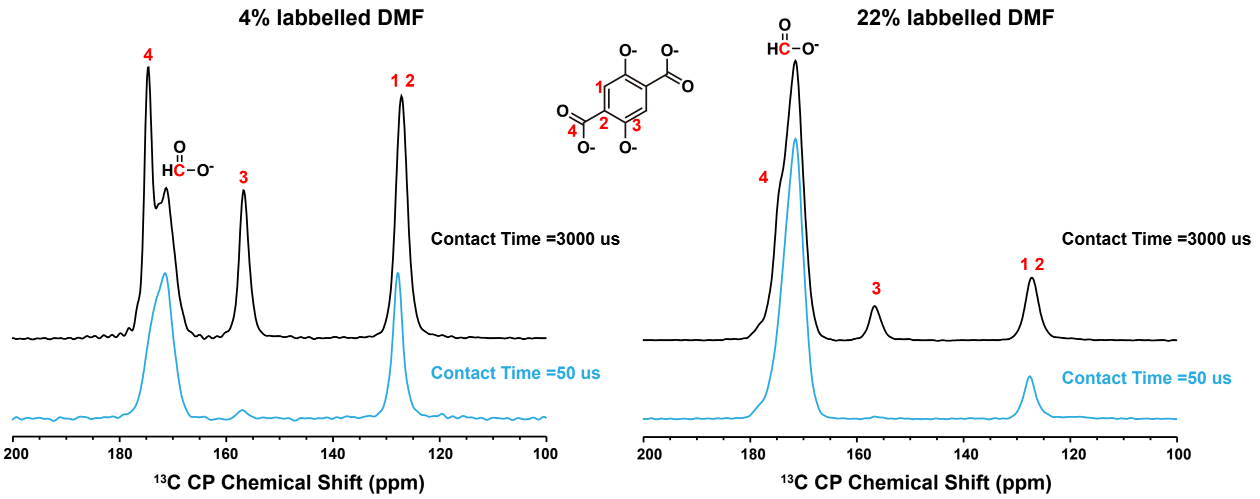

Supplementary Figure 10. ^{13}C CPMAS spectra with a short contact time (CT=50 μs, blue line, showing the carbons directly bonded by H) and with a long contact time (CT=3000 μs, black line, showing all carbon sites) on 0.23D MOF samples synthesized in 4% carbonyl-^{13}C labeled DMF or 22% carbonyl-^{13}C labeled DMF.

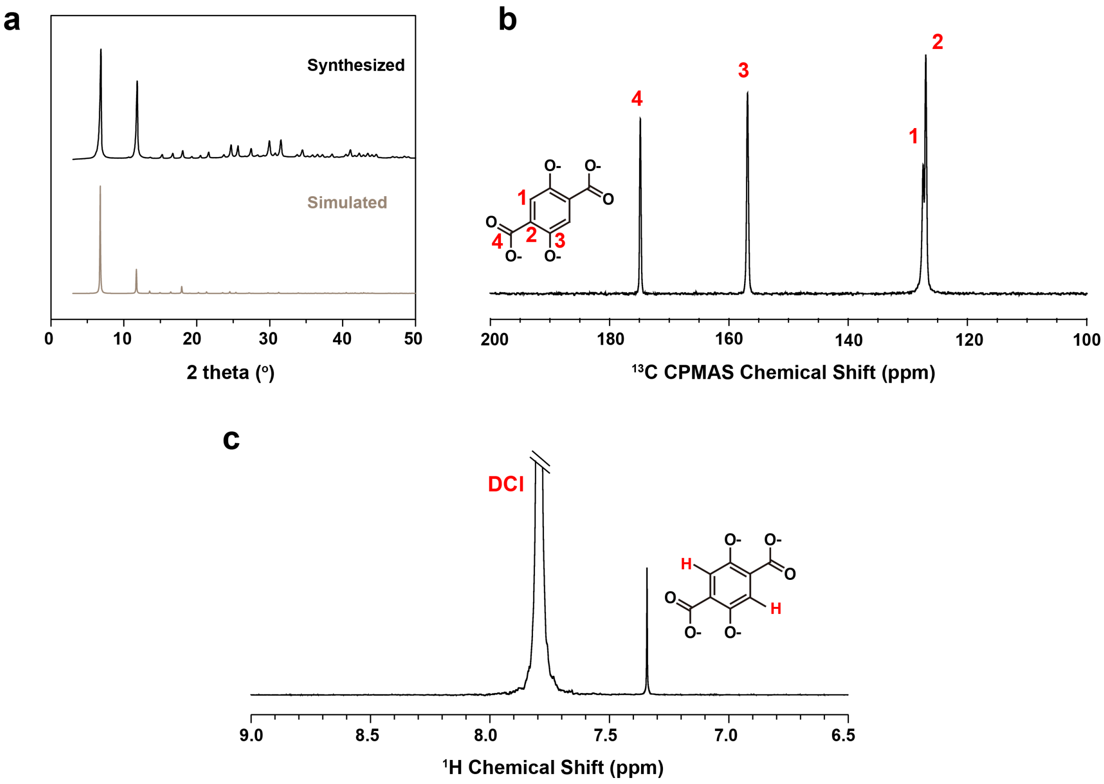

Supplementary Figure 11. (a) PXRD pattern and (b) ^{13}C CPMAS spectrum of Mg-MOF-74 synthesized in THF solvent (metal-to-linker ratio = 2:1). (c) ^{1}H solution-state NMR spectrum of digested Mg-MOF-74 synthesized in THF solvent. No formate signal is observed.

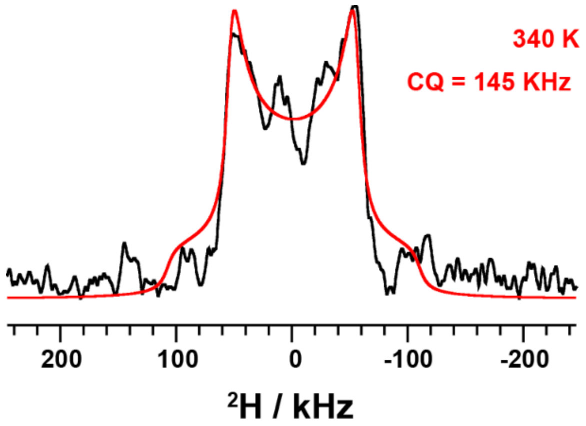

Supplementary Figure 12. Experimental (black line) and fitted (red line) ^{2}H spectra of the d_1-formate in defective Mg-MOF-74. The ^{2}H NMR spectrum at 340 K has approximately the same quadrupolar splitting of 145 kHz as that obtained at 300 K (Figure 2a), indicating the formate is rigid.

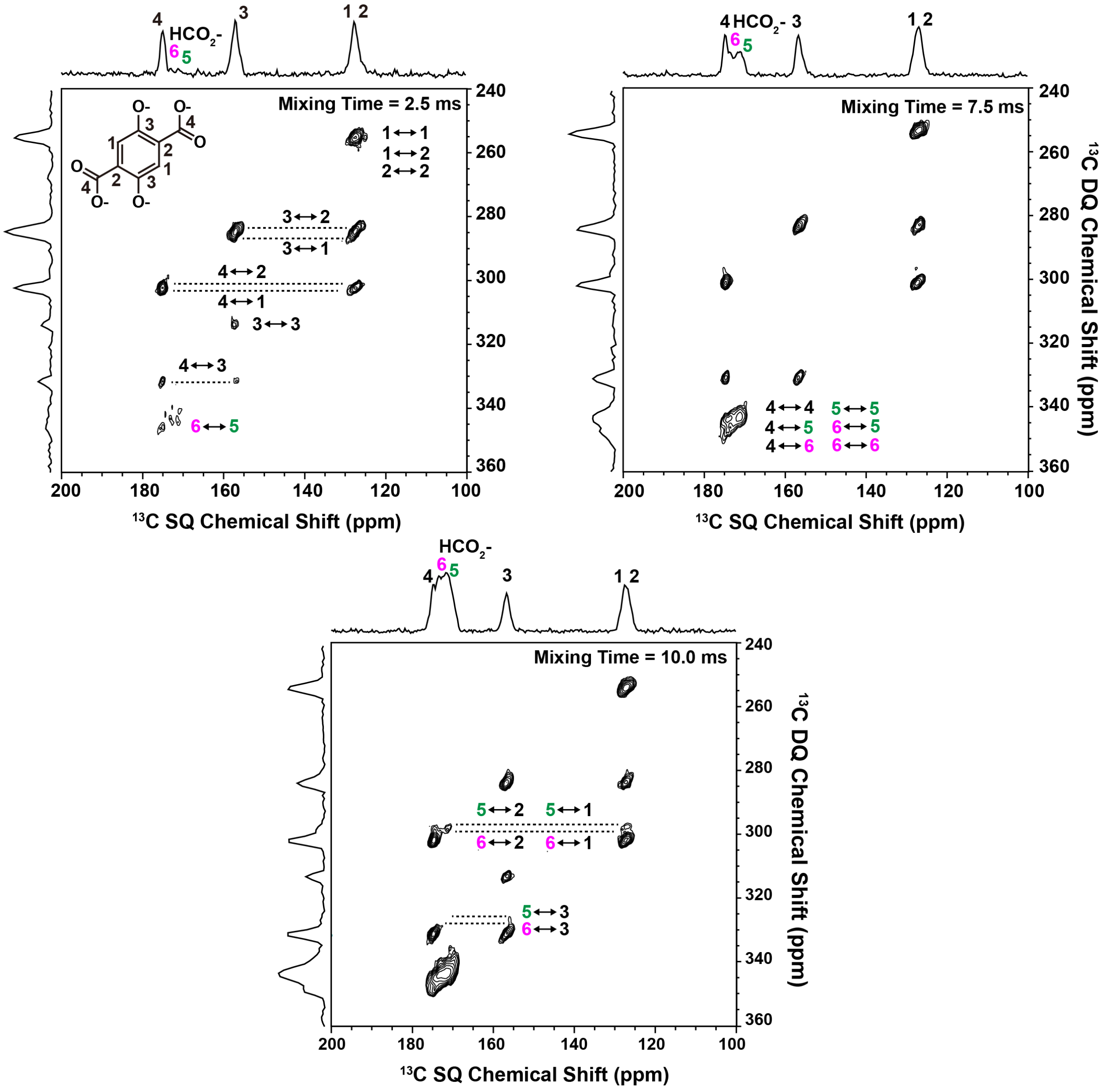

Supplementary Figure 13. 2D ^{13}C-^{13}C double-quantum single-quantum (DQ-SQ) correlation spectra of defective 0.23D sample recorded at 100 K with different mixing times. The correlation peaks are marked with numbers.

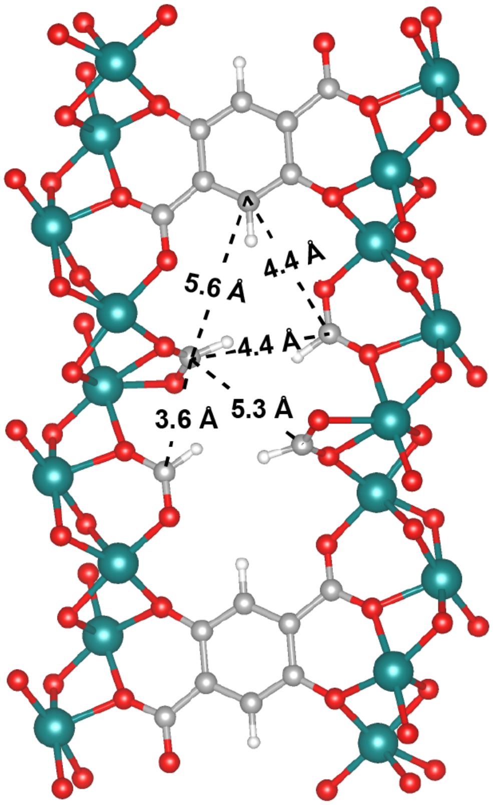

Supplementary Figure 14. Defective Mg-MOF-74 structure optimized by DFT calculations. Blue, red, grey and white spheres represent Mg, O, C and H atoms, respectively. The distances between different carbons are indicated. Source data are provided as a Source Data file.

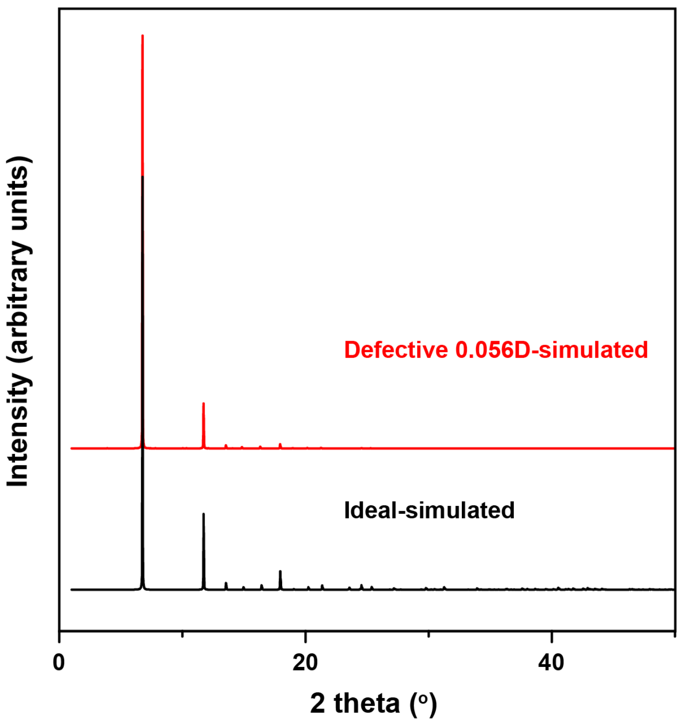

Supplementary Figure 15. PXRD patterns of ideal and defective 0.056D Mg-MOF-74 samples based on DFT simulations. Source data are provided as a Source Data file.

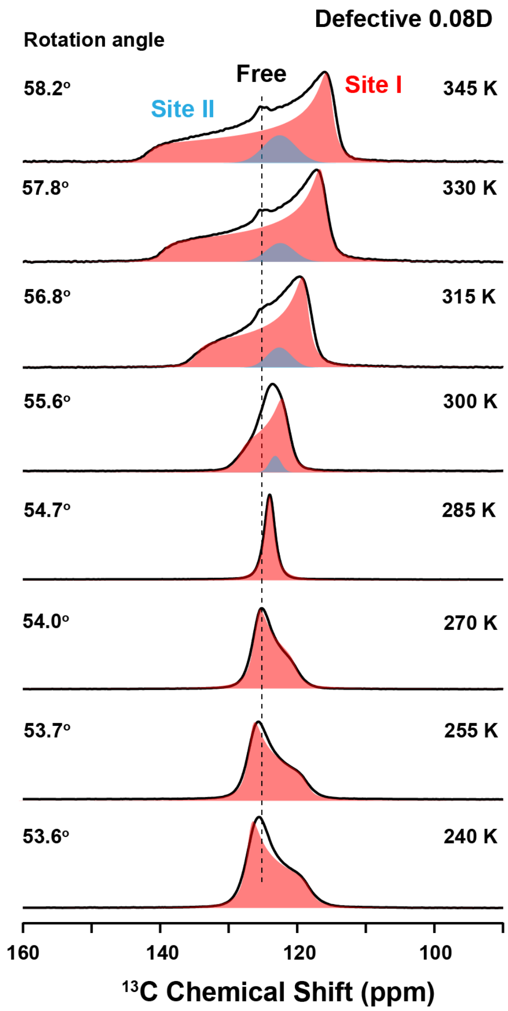

Supplementary Figure 16. In situ static ^{13}C NMR of ^{13}C-enriched CO_2 in defective Mg-MOF-74 under variable temperatures. The red patterns are the simulated CSA lineshapes of CO_2 uniaxial rotation with corresponding rotational angles (attributed to the adsorption at site I). The blue patterns are Gaussian lineshapes for the CO_2 adsorption at site II. A vertical line is placed at 124.5 ppm to reference the isotropic chemical shift of free CO_2.

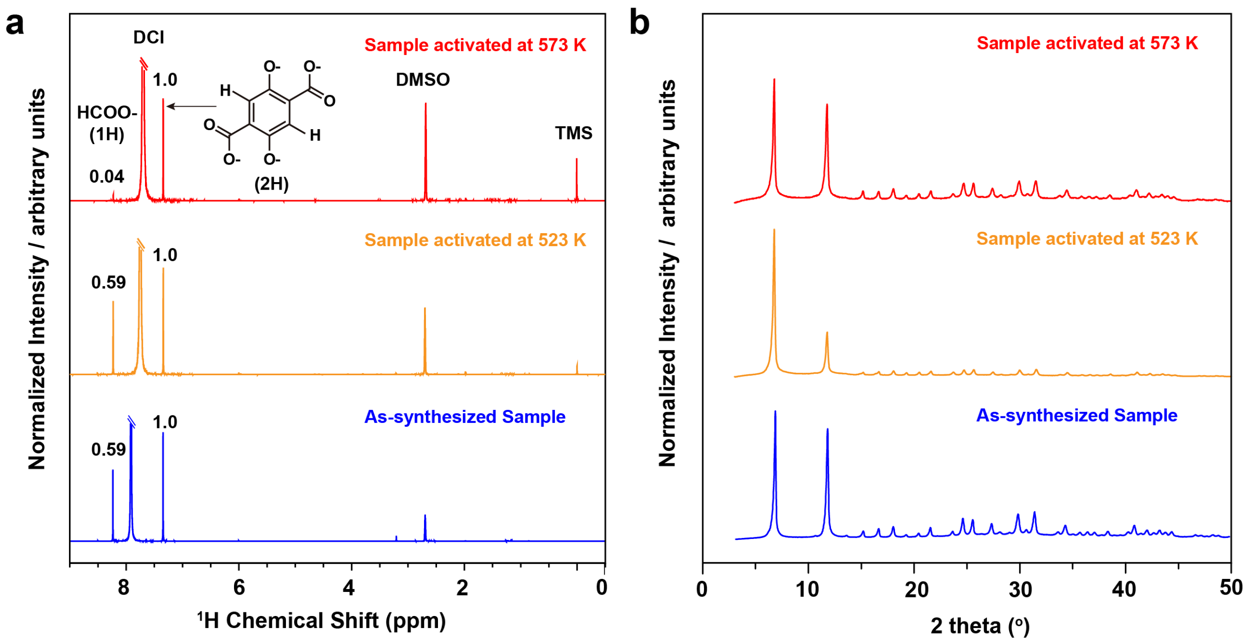

Supplementary Figure 17. (a) ^{1}H solution-state NMR spectra of digested 0.23D sample. The sample was treated in different conditions. The 8.2-ppm peak of formate peak decreases in intensity significantly when the sample was activated at 573 K. (b) PXRD patterns of 0.23D sample under different treatments. The 0.23D sample maintains the crystallinity even after activation at 573 K.
