# Supplementary material for: Solvent-derived defects suppress adsorption in MOF-74
Source: Nat Commun. 2023 Apr 25;14:2386. doi: 10.1038/s41467-023-38155-8 (PMC10130178; doi:10.1038/s41467-023-38155-8)
Supplement: Supplementary file 3 — Source Data [file 41467_2023_38155_MOESM3_ESM.zip › Source Data/File_List.pdf]

File Name	Content
MgMOF74-i.cif	Intact MOF-74 structure optimized in CP2K. This structure is replicated twice in the z-axis for NVT-MD/GCMC simulations.
MgMOF74-d.cif	Defective MOF-74 structure optimized in CP2K. The defect concetration x is 0.056 in the formula of Mg2(dobdc)1-x(HCO2)4x.
in.nvt-i	Lammps input file for NVT-MD/GCMC simulations of the intact MOF
in.nvt-d	Lammps input file for NVT-MD/GCMC simulations of the defective MOF
data.gcmc.i	Lammps input file for NVT-MD/GCMC simulations of the intact MOF, which contains configuration and topology of the simulated system
data.gcmc.d	Lammps input file for NVT-MD/GCMC simulations of the defective MOF which contains configuration and topology of the simulated system
CO2.txt	Lammps input file for NVT-MD/GCMC simulations, which includes the configuration of carbon dioxide to be inserted during NVT-MD/GCMC simulations
d-lat-nmr.inp	Input file for NMR calculations in CP2K (The calculations can be conducted by the command "cp2k.ssmp -i ./d-lat-nmr.inp 1>nmr.out 2>nmr.err".)
